# Modeling and Experiment of the Critical Depth of Cut at the Ductile–Brittle Transition for a 4H-SiC Single Crystal

**DOI:** 10.3390/mi10060382

**Published:** 2019-06-07

**Authors:** Peng Chai, Shujuan Li, Yan Li

**Affiliations:** School of Mechanical and Precision Instrument Engineering, Xi′an University of Technology, No. 5 South Jinhua Road, Xi’an 710048, Shaanxi, China; chaipeng@stu.xaut.edu.cn

**Keywords:** 4H-SiC, critical depth of cut, Berkovich indenter, cleavage strength, nanoscratching

## Abstract

In this paper, a theoretical model of the critical depth of cut of nanoscratching on a 4H-SiC single crystal with a Berkovich indenter is proposed, and a series of scratch tests in a nanomechanical test system was performed. Through nanoindentation experimentation on fused quartz, the Berkovich indenter nose radius was indirectly confirmed using least squares. The range of critical depths of cut at the ductile–brittle transition was obtained by SEM observation, and the size of cracks was amplified with increasing scratching depth. The theoretical result of the critical depth of cut at the ductile–brittle transition for a 4H-SiC single crystal is 91.7 nm, which is close to the first obvious pop-in point of the relation curve between tangential force and lateral displacement. Repeated experimental results show good consistency and good agreement with other references.

## 1. Introduction

With excellent electronic characteristics such as a large band gap, high critical breakdown strength, high electronic saturation rate, high thermal conductivity, and high irradiation resistance, silicon carbide (SiC) has become an outstanding representative of the third generation of semiconductor materials and has been increasingly widely applied in a variety of fields, including computer, aviation, power, and nuclear energy development [1,2,3,4]. In addition, it is an attractive option to use SiC to produce space mirrors and large ground-based reflectors thanks to its remarkable advantages of large stiffness, small thermal deformation coefficient, and good thermal stability [5]. However, it is difficult to obtain SiC parts with high forming accuracy and ideal machined surface quality due to its characteristics of high hardness (with a Mohs hardness between 9.0 and 9.5) and brittleness [6,7].

The traditional cutting force models tend to ignore the effect of elastic recovery on the macroprocessing of ductile materials. However, numerous studies [8,9,10] have shown that the role of elastic recovery is significant in the micro–nano-machining of brittle materials. Wasmer et al. [11] proposed a typical scratch pattern for brittle materials using an increasing load along a scratch path that is divided into five stages: elastic regime, ductile regime, subsurface crack regime, surface crack regime, and micro-abrasive regime. Elastic and ductile deformations were observed before the ductile regime, and brittle fractures appeared and began to dominate the latter deformation with increasing cutting force. Therefore, there is a cutting depth, referred to as the critical depth of cut, where the ductile region transitions to the brittle region. Lawn et al. [12] established a microfracture model under the point indentation of brittle materials consisting of the following processes: (1) the sharp indenter induces a zone of elastic and ductile deformation around the contact point; (2) a median crack suddenly initiates below the contact point; (3) the median crack stably extends with increasing indenter load; (4) the median crack begins to close during the initial unloading process; (5) lateral cracks begin to appear due to residual stress; and (6) lateral cracks continue to extend and cause chipping.

Methods such as grinding and chemical mechanical polishing are used in traditional processing and are characterized by low production efficiency, high production cost, and, particularly, surface damage caused by the contamination of the polishing slurry [13]. In 1951, researchers found that hard and brittle materials show the characteristics of ductile removal under certain processing conditions [14]. Since the 1990s, researchers have conducted many studies into removal mechanisms in silicon carbide ductile regime processing, such as ductile regime grinding [7,15], ductile regime laser-assisted processing [16], ductile regime diamond cutting [17], ductile regime diamond wiresaw [18], and ductile domain ultrasonic-assisted processing [19]. These studies indicated that in the course of ductile regime processing, the chip is removed by ductile deformation, causing no damage or cracks to the machined surface of the workpiece, and the surface processing quality can be maintained [20,21,22]. The critical depth of cut at the ductile–brittle transition, the maximum cutting depth where no cracks appeared on the surface or subsurface of the sample, is a fundamental parameter of all methods of ductile regime processing. A formula was obtained by Bifano according to Griffith’s principle through a quasi-static scratch test of several kinds of common brittle materials [23]:

(1) $$d_{c}=\alpha \left(\frac{E}{H}\right){\left(\frac{K_{C}}{H}\right)}^{2})$$

where *α* is a constant, *E* is the elasticity modulus, *H* is the hardness, and *K*_c_ is the fracture toughness. This formula was amended by later scholars; for example, Gaobo’s study on the critical depth of cut of 6H-silicon carbide indicated that the experimental results were not in line with the calculation of Formula (1) and the amended constant *α* [24].

Based on the above, most research has focused on the critical depth of cut using Griffith’s principle and experimental method, which is based on cracks in the ductile extension which would have an influence on the performance of devices. With the development of nanotechnology, particularly the scanning electron microscope (SEM) and nanomechanical testing technology, a number of powerful tools has been provided to investigate the properties of silicon carbide at the nanoscale. This paper proposes a method considering the elastic recovery of the workpiece surface in nanoscratching in order to obtain the critical depth of cut for SiC using scratching stress and cleavage strength.

## 2. Modeling

### 2.1. Modeling of the Indenter Structure

**Figure 1:**
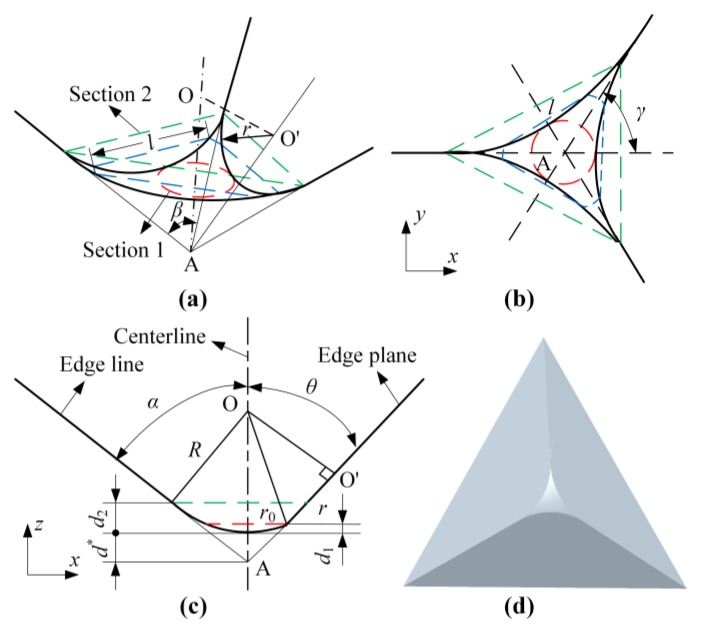
Dimension parameters and geometric shape of the Berkovich indenter: (**a**) model diagram, (**b**) top view, (**c**) side view, and (**d**) 3-D solid model.

The indenter tip shape greatly affects nanoscratching results, and there is no ideal Berkovich indenter due to the limitations of processing conditions. In addition, the indenter continuously wears as it is working; therefore, the indenter tip shape is different for every test. The geometric shape and dimension parameters of a Berkovich indenter are shown in Figure 1. Assuming that a Berkovich indenter is a combination of a sphere and a triangular pyramid [25], the tip can be divided into three parts: the sphere (from Section 1 to the vertex of the nose in Figure 1a), the transition (from Section 1 to Section 2), and the pyramid (above Section 2). The following equations can be obtained according to the geometric relations:

(2) $$d^{*}=\frac{R}{\sin\alpha }-R$$

(3) $$d_{2}=R\left(1-\sin\alpha \right)$$

(4) $$r_{0}{}^{2}=R^{2}-{\left(R-d_{1}\right)}^{2}$$

(5) $$\tan\theta =\frac{r_{0}}{d^{*}+d_{1}}$$

(6) $$r=\sqrt{R^{2}-{\left(R+d*\right)}^{2}\sin^{2}\theta }$$

(7) $$l=2\sqrt{r^{2}-{\left(r-\frac{d-d_{1}}{\cos\theta }\right)}^{2}}$$

where *R* is the indenter nose radius, d* is the distance from the nose vertex to the top of the ideal indenter, *α* is the angle between the edge line and the centerline, *θ* is the angle between the edge plane and the centerline, *d*_1_ is the distance from Section 1 to the nose vertex, *d*_2_ is the distance from Section 2 to the nose vertex, *r*_0_ is the radius of Section 1, *r* is the circular arc radius of the intersection between the sphere and the triangular pyramid, and *l* is the length of the intersection between the section which is normally aligned to the centerline and the edge plane in the transition part.

The normal projected area of the different indenter heights can be calculated using Equation (8).

(8) $$A_{p}=\{\begin{array}{ll} \pi [R^{2}-{\left(R-d\right)}^{2}] & \left(d\leq d_{1}\right)\\ \pi \tan^{2}\alpha {\left(d+d^{*}\right)}^{2}-\frac{\arcsin\frac{l}{2\tan\alpha (d+d^{*})}}{60}\cdot \pi \tan^{2}\alpha {\left(d+d^{*}\right)}^{2}+\frac{3l}{2}\tan\alpha (d+d^{*}) & \left(d_{1}<d<d_{2}\right)\\ \frac{3\sqrt{3}}{4}\tan^{2}\alpha {\left(d+\frac{R}{\sin\alpha }-R\right)}^{2}. & \left(d\geq d_{2}\right) \end{array}$$

### 2.2. Modeling of the Critical Depth of Cut

Based on the traditional cutting force model, indentation model, and scratch pattern, a new method considering the elastic recovery was designed and two assumptions were proposed: (1) As a rigid body, the indenter does not deform; however, it wears during the process of scratching. (2) The motion of the indenter is quasi-static. According to the characteristics of deformation, the process of scratching can be divided into three stages: the elasticity leading stage, the ductility leading stage, and the brittleness leading stage [26].

In the elasticity leading stage, the force applied on the indenter consists of an elastic restoring force, an adhesive force, and a frictional force. The elastic restoring force is a reactive force applied on the indenter caused by the elastic deformation of the part. The adhesive force between the two solids (i.e., indenter and part) is complex [27]; therefore, a simplified approach that combines the adhesive and frictional forces is used in this study. All forces are decomposed into normal and tangential forces:

(9) $$\begin{array}{l} F_{en}=K_{1}A_{1}\\ F_{et}=\mu K_{1}A_{1} \end{array}$$

where *K*_1_ is the average contact pressure between the indenter and workpiece, *A*_1_ is the projected area of the contact surface between the indenter and part ($A_{1}=3\sqrt{3}{\left(d+d^{*}\right)}^{2}\tan^{2}\theta$), and *μ* is the frictional and adhesive coefficient.

The study by Son et al. [28] showed that the minimum cutting depth producing chips can be expressed as

(10) $$d_{m}=R\left(1-\cos\left(\frac{\pi }{4}-\frac{\beta }{2}\right)\right)$$

where *β* is the friction angle.

**Figure 2:**
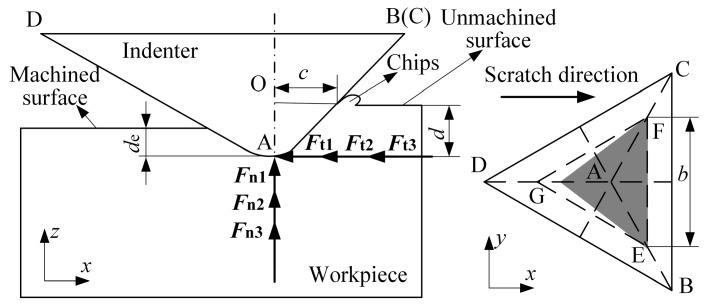
Force analysis model in the ductility leading stage.

In the ductility leading stage, the force applied on the indenter consists of an elastic restoring force, frictional and adhesive forces, and cutting deformation force. The force analysis is illustrated in Figure 2. The frictional and adhesive forces consist of two parts: one caused by chip formation and one caused by elastic recovery. The cutting deformation force, which is a reaction force applied on the indenter caused by the deformation of the part, can be separated into a chip formation force and a plowing force. However, the plowing force can be ignored in this model since it is much weaker than the chip formation force [29]. All forces are decomposed into normal and tangential forces:

(11) $$\begin{array}{l} F_{dn}=F_{dn1}-F_{dn2}+F_{dn3}\\ F_{dt}=F_{dt1}+F_{dt2}+F_{dt3} \end{array}$$

where *F_dn_*_1_ is the normal force component caused by the elastic restoring force, *F_dn_*_2_ is the normal force component caused by the frictional and adhesive forces, *F_dn_*_3_ is the normal force component caused by the chip formation force, *F_dt_*_1_ is the tangential force component caused by the elastic restoring force, *F_dt_*_2_ is the tangential force component caused by the frictional and adhesive force, and *F_dt_*_3_ is the tangential force component caused by the chip formation force.

The normal force components can be calculated using Equation (12).

(12) $$\begin{array}{l} F_{dn1}=K_{1}S_{1}\\ F_{dn2}=\mu (K_{1}+K_{2})S_{2}\sin\theta \\ F_{dn3}=K_{2}S_{2}\sin\theta \end{array}$$

where *K*_2_ is the cutting deformation contact stress, *S*_1_ is the projected area given by the shaded area in Figure 2, and *S*_2_ is the contact area between the region of ductile deformation, which is the area from the unmachined surface to the machined surface.

The areas *S*_1_ and *S*_2_, respectively, are

(13) $$\begin{array}{l} S_{1}=\sqrt{3}[(d_{e}+d^{*})\tan\alpha +(d+d^{*})\tan\theta ](d+d^{*})\tan\theta \\ S_{2}=\frac{\left(d-d_{e}\right)}{2\cos\theta }[\sqrt{3}(d+d^{*})\tan\theta +\sqrt{3}(d_{e}+d^{*})\tan\theta ] \end{array}$$

where *d*_e_ is the part elastic recovery depth and is equal to the height difference between the scratching and residual depths. Note that the elastic recovery depth is not constant and increases linearly as the scratching depth increases [20].

The tangential force components are

(14) $$\begin{array}{l} F_{dt1}=K_{1}S_{2}\cos\theta \\ F_{dt2}=\mu (K_{1}+K_{2})S_{2}\cos\theta +\mu K_{1}S_{1}\\ F_{dt3}=\frac{F_{dn3}}{\tan\theta } \end{array}$$

The normal and tangential forces, respectively, are

(15) $$\begin{array}{l} F_{dn}=K_{1}S_{1}-\mu (K_{1}+K_{2})S_{2}\sin\theta +K_{2}S_{2}\sin\theta \\ F_{dt}=K_{1}S_{2}\cos\theta +\mu (K_{1}+K_{2})S_{2}\cos\theta +\mu K_{1}S_{1}+K_{2}S_{2}\cos\theta \end{array}$$

In the brittleness leading stage, the average contact pressure and the cutting deformation contact stress show a zigzag change due to crack propagation and pop-in debris.

It is difficult to control the depth of processing, but controlling the cutting force, especially the normal force, is relatively easy, no matter whether ultra-precision grinding or single point diamond cutting is used. Therefore, the cutting force model of this section can be used to control the cutting depth through the cutting force.

The dislocation will appear when the part undergoes extrusion deformation [30]. The appearance of a cleavage crack will occur when one side’s tensile stress reaches the limit under the action of the applied force. The theoretical cleavage strength can be expressed by [31]

(16) $$\sigma_{c}=\frac{1}{2}\sqrt{\frac{E\gamma }{a}}$$

where *E* is elastic modulus, *γ* is surface energy per unit area, and *a* is the interplanar spacing. In the scratching process, the maximum stress in the part’s machined surface is located at the tip of the indenter. If the maximum stress is less than the cleavage strength, no cracks will occur on the surface or subsurface of the part. The maximum stress is [32]

(17) $$P_{0}=\frac{3}{2}K_{1}$$

The parameter *K*_1_ is obtained from Equation (15).

(18) $$K_{1}=\frac{F_{\mathrm{dn}}(\mu S_{2}\cos\theta +S_{2}\cos\theta )-F_{\mathrm{dt}}(S_{2}\sin\theta -\mu S_{2}\sin\theta )}{\left(S_{1}-\mu S_{2}\sin\theta )(\mu S_{2}\cos\theta +S_{2}\cos\theta )-(S_{2}\cos\theta +\mu S_{2}\cos\theta +\mu S_{1})(S_{2}\sin\theta -\mu S_{2}\sin\theta \right)}$$

The critical condition of brittle materials during the scratching process is

(19) $$\sigma_{c}=\frac{3}{2}K_{1c}$$

where *K*_1*c*_ is the critical average contact pressure.

## 3. Experimental Setup

A commercial wafer of 4H-SiC single crystal, grown using the physical vapor transport method, was supplied by Shanghai Institute of Optics and Mechanics Chinese Academy of Sciences. The 4H-SiC wafer was cut to the size of 10 mm × 10 mm by a laser cutting machine and its thickness was 0.5 mm. It was measured using a scanning electron microscope (SEM), and the surface roughness was found to be less than 1 nm after a polishing treatment. All experiments conducted in this paper were conducted on the (0001) plane. A diamond Berkovich indenter with an angle between the edge plane and the centerline of 65.27° was used in this study, and a standard fused quartz sample was employed to indirectly measure the nose radius of the indenter.

**Figure 3:**
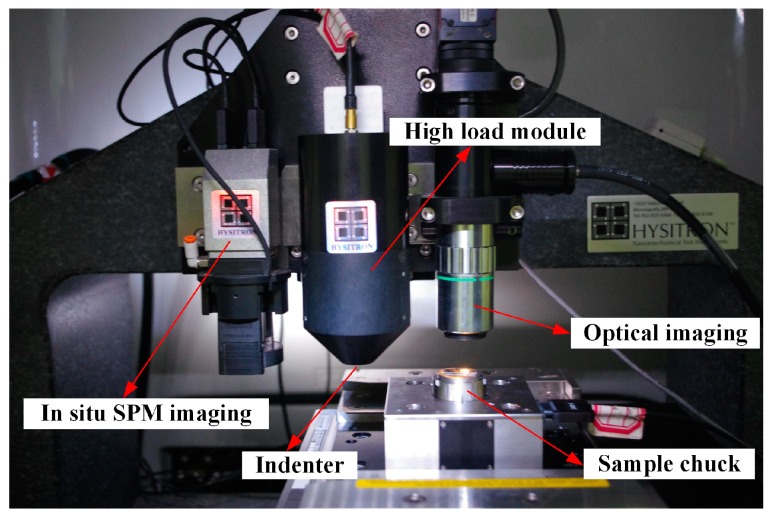
Core components of the TI 950 Triboindenter.

A laser cutting machine (HGLaser LCC0130-CO2, HGTECH, Wuhan, China) was used to prepare the sample used in this study. A nanomechanical test instrument (TI 950 Triboindenter, Hysitron, Minneapolis, MN, USA), with load sensitivity less than 30 nN and displacement sensitivity less than 0.2 nm, was used to scratch the sample surface and record the information of the scratching depth, tangential force, normal force, and time and to obtain in situ scanning probe microscopy (SPM) images. The instrument is shown in Figure 3. Scanning electron micrographs of the scratch were generated using a foucused ion beam-scanning electron microscope (FIB-SEM) system (Helios NanoLab 600i, FEI, Hillsboro, OR, USA).

The experiments consisted of two parts, namely, the indentation and scratch experiments. In order to determine the nose radius of the indenter, nine indents were made at maximum load capacity from 20 to 180 mN at a constant interval on the standard fused quartz sample surface, and a constant loading rate was used in the process of the experiment. All experiments were performed with a 10 s holding time at room temperature.

The scratch process on the commercial wafer of the 4H-SiC single crystal included three stages: (1) the pre-scan stage, (2) the scratching stage, and (3) the post-scan stage. In the first stage, the sample surface morphology information, such as surface roughness and sample inclination angle, were obtained when the indenter was scanned on the sample surface with a constant load of 0.1 mN. In the scratching stage, the indenter load was increased linearly from 0 to 80 mN while the sample table moved at a constant rate, and the length of the scratch on the sample surface was 250 μm. In the final stage, the indenter scanned backwards with a constant load of 0.1 mN to obtain the scratched surface morphology information. The scratch test parameters are shown in Table 1. The scratched surface topography imaging was delivered by dual piezo scanners in the in situ SPM imaging system.

A FIB-SEM system was used to measure and evaluate the deformation characteristics of scratches and cracks on the samples immediately after the nanoscratching tests.

## 4. Results, Analysis, and Discussion

### 4.1. Determination of the Indenter Nose Radius

**Figure 4:**
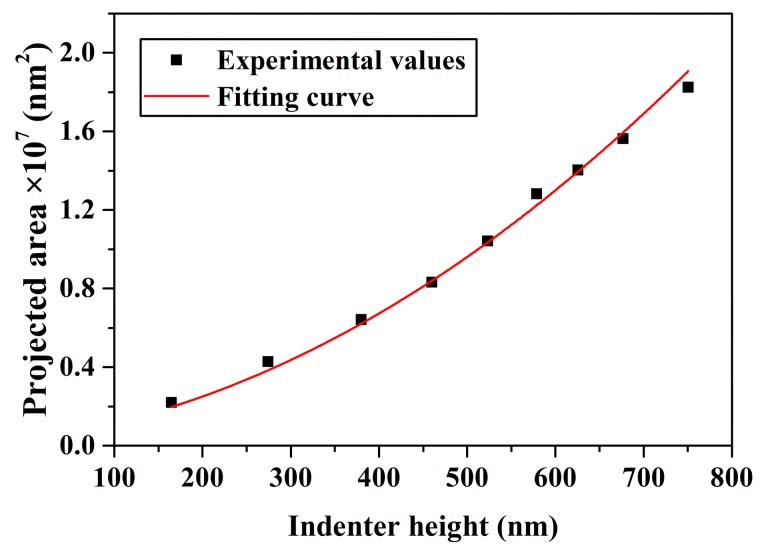
Relationship between the projected area and indenter height.

An indirect method to compare the theoretical projected area, which is a function of *R* and *d*, with the area function acquired through nanoindentation on the standard fused quartz sample was used to determine the numerical value of *R*. The hardness, *H*, can be expressed as [33]

(20) $$H=\frac{F_{\max}}{A_{p}}$$

where *F*_max_ is the maximum load. For standard fused quartz, the hardness is 9.5 GPa. For the Berkovich indenter used in this study, *α* = 77.3°, *β* = 57.64°, *θ* = 65.27°, and *γ* = 60°. Table 2 shows the indenter height and projected area for a variety of maximum loads. Using least squares, the projected area was related to the indenter height by $A_{p}=25.58\times {\left(123.8+d\right)}^{2}$, as shown in Figure 4. The indenter nose radius was calculated as *R* = 4952 nm via Equation (8).

### 4.2. Analytic Surface Morphology

**Figure 5:**
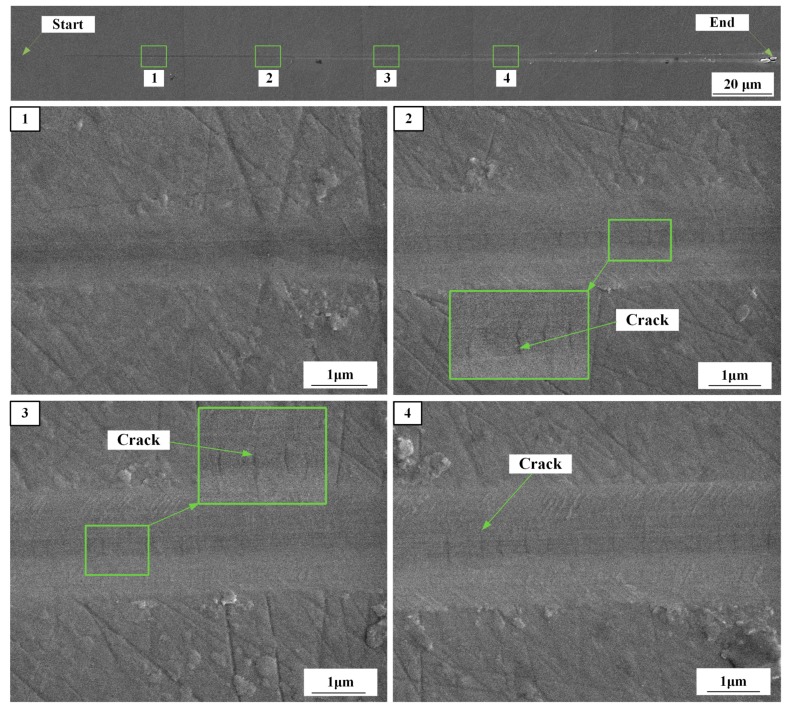
Full view and enlarged image of a scratch using SEM.

The surface morphology of the scratch is shown in Figure 5. It was observed in the enlarged image that material is removed but no cracks are formed in the surface at Position 1. There are cracks at the bottom of the scratch at Positions 2 to 4; these are perpendicular to the scratch motion and are the result of median crack closure and lateral crack growth due to the residual stress caused by the indenter. The size of the cracks was amplified with increasing scratching depth. Subsurface cracks were revealed with the scanning electron microscope. Therefore, the results show that the ductile–brittle transition of 4H-SiC is located before Position 2, as shown in Figure 5, and the corresponding scratch length ranges from 0 to 80 μm, with the scratching depth ranging from 0 to 120 nm.

### 4.3. Comparison of the Critical Depth of Cut between Simulation and Experiments

**Figure 6:**
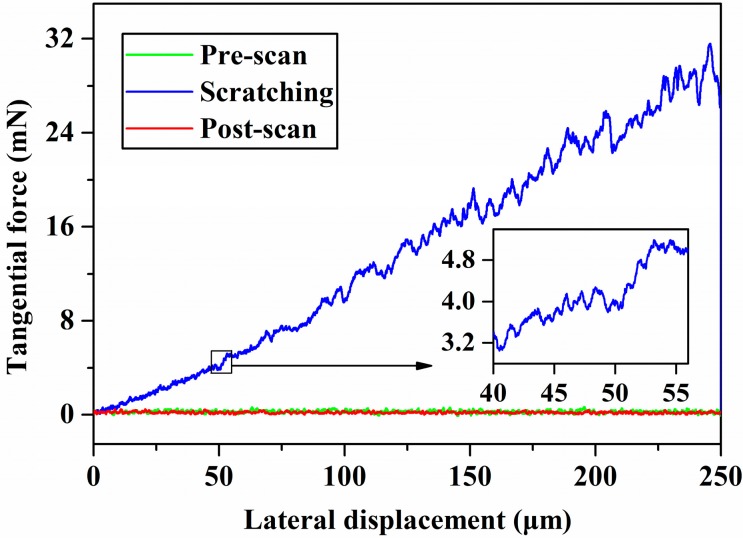
Tangential force as a function of lateral displacement.

**Figure 7:**
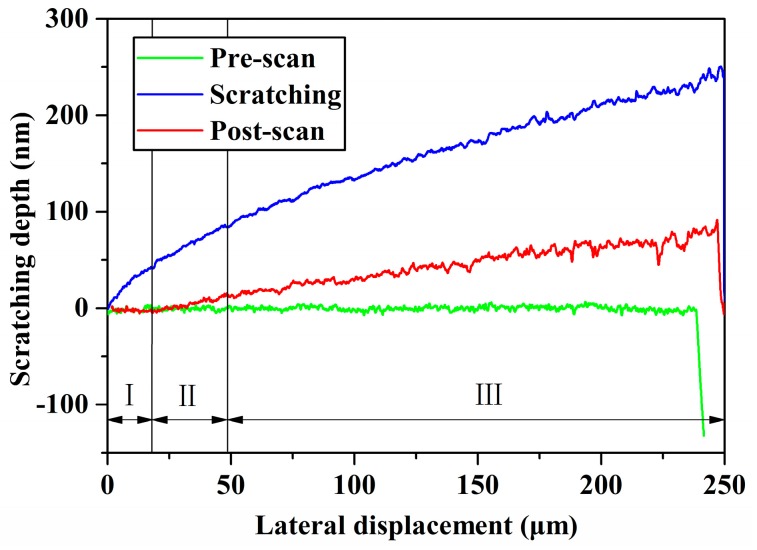
Scratching depth as a function of lateral displacement.

The experimental data were obtained using a two-dimensional three-plate capacitive sensor and a TI 950 piezoelectric ceramic. The experimental results are shown in Figure 6; Figure 7. Figure 6 shows the tangential force as a function of the lateral displacement. Figure 7 shows the scratching depth as a function of the lateral displacement.

According to the theory mentioned in Section 2.2, the whole scratch can be divided into three stages: Ⅰ, standing for the elasticity leading stage; Ⅱ, standing for the ductile leading stage; and Ⅲ, standing for the brittleness leading stage, as shown in Figure 7. The minimum scratching depth is 40 nm and the elastic recovery depth/scratching depth ratio is 0.77 through an analysis of the scratching depth versus lateral displacement curve. In the elasticity leading stage, i.e., where the scratching depth is less than 40 nm, the experimental data were plugged into Equation (9), and we received a frictional and adhesive coefficient of *μ* = 0.31; this is much larger than the frictional coefficient, which is equal to 0.05 [34].

**Figure 8:**
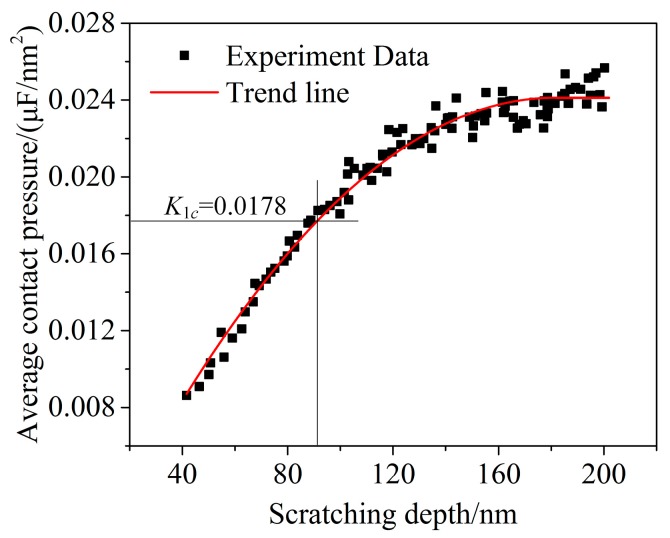
Average contact pressure as a function of scratching depth.

**Figure 9:**
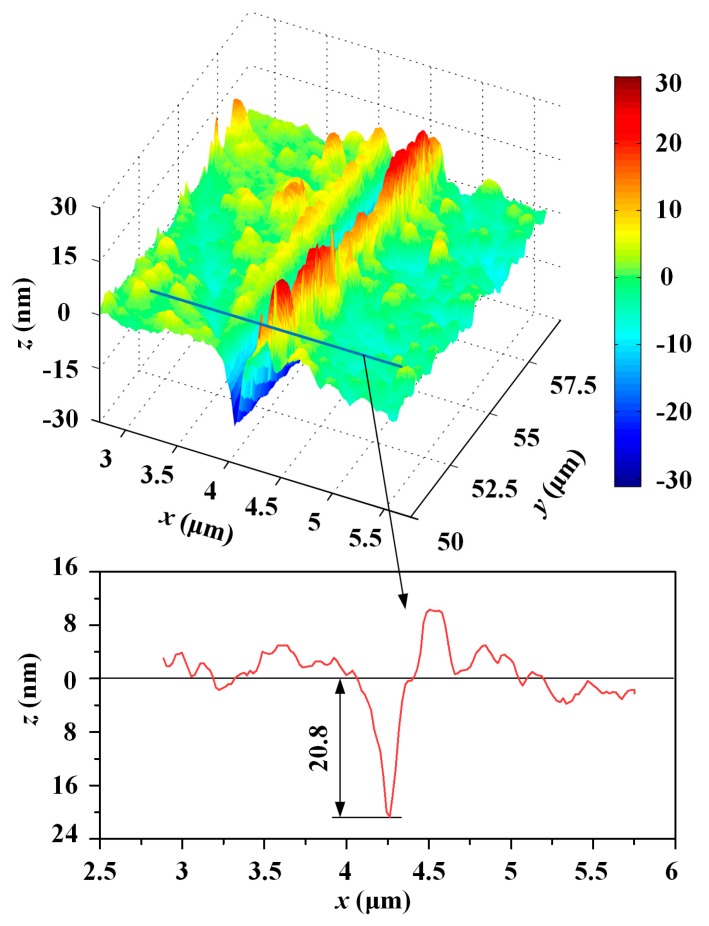
In situ SPM images.

In the ductile leading stage, i.e., where *d* ≥ 40 nm, the average contact pressure was computed via Equations (13) and (18) and is shown in Figure 8. The cleavage strength of silicon carbide is 26.7 Gpa [35]. The critical average contact pressure is 17.8 Gpa via Equation (19). According to the relationship between the average contact pressure and the scratching depth, the critical depth of cut of 4H-SiC was determined to be 92 nm. Extension of the crack can cause a drastic change in tangential force and the appearance of a pop-in phenomenon. The first pop-in point of the relation curve between tangential force and lateral displacement appears where the scratching depth is about 90 nm, and it is very close to the theoretical calculation results. The in situ SPM images where the lateral displacement ranged from 50 to 60 μm, including the critical depth of cut, indicate that the residual depth, when located in the critical depth of cut, is 20.8 nm, as shown in Figure 9. The two aforementioned scratch tests were repeated in order to exclude the contingency of a single-pass test. The same process was used to handle the test data, and the results are shown in Table 3. This result shows good agreement with other references, as shown in Table 4.

The sources of error in this study are as follows: (1) The frictional and adhesive coefficient between the indenter and sample surface is not a constant in the process of scratching when loaded linearly [41], but it was simplified to a constant in this study. (2) The wear of the indenter was ignored. (3) The impact of defects in the crystal, such as microtubules, dislocations, and stacking faults, was also ignored. (4) Although the roughness of the sample surface was less than 1 nm, it still has a considerable influence in nanoscale experiments.

## 5. Summary and Conclusions

A theoretical model of the critical depth of cut of nanoscratching on a 4H-SiC single crystal with a Berkovich indenter was proposed, and a scratch test in a nanomechanical test system was conducted. The following conclusions can be drawn from this study:

(1) Based on an analysis of the nanoindentation and typical scratch model, a new model of the critical depth of cut of nanoscratching on a 4H-SiC single crystal with a Berkovich indenter was established.

(2) The radius of the Berkovich indenter nose was indirectly confirmed by a nanoindentation experiment, and the range of cracks on the scratched surface was verified by SEM images.

(3) The change in the sample surface in the scratching process was revealed through the average contact pressure. The theoretical result of the critical depth of cut at the ductile–brittle transition for a 4H-SiC single crystal was obtained; it is close to the first obvious pop-in point of the relation curve between tangential force and lateral displacement, and this result shows good agreement with other references.

## Figures and Tables

**Table 1:** Scratch test parameters.

Test Parameters	Unit	Values
Pre-scan/post-scan load	mN	0.1
Loading range	mN	0.1–80
Scratch length	μm	250
Scratch velocity	μm/s	4

**Table 2:** Indenter height and contact area for different loads.

Load (mN)	Indenter Height (nm)	Contact Area (nm^2^)
20	164.5	2.2053 × 10^6^
40	274.2	4.2905 × 10^6^
60	379.6	6.4158 × 10^6^
80	460.1	8.3211 × 10^6^
100	522.9	1.0426 × 10^7^
120	578.6	1.3032 × 10^7^
140	625.4	1.4037 × 10^7^
160	676.5	1.5642 × 10^7^
180	750.4	1.8247 × 10^7^

**Table 3:** Repeated test results.

Test Number	Critical Depth of Cut (nm)
1	92
2	93
3	90
Average value	91.7

**Table 4:** Indenter height and contact area for different loads.

Critical Depth of Cut (nm)	Material	Speed (mm/s)	Tip Radius (μm)	Refs.
75	6H-SiC	0.01	0.94	[36]
95	4H-SiC	0.001	5	[37]
<100	6H-SiC	150	0.05	[38]
70	6H-SiC	82.5	0.05	[39]
<60	6H-SiC	4.5	800	[40]
